# Dr. Navnendra Mathur - An astute clinician and endearing friend

**Published:** 2009

**Authors:** Rakesh Bhargava

**Affiliations:** *Professor and Head, Department of Orthopedics, SMS Medical College, Jaipur* E-mail: drrakeshbhargava@hotmail.com


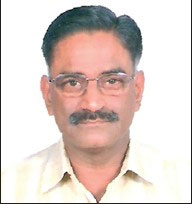


**Dr. Navnendra Mathur (28.09.1951 - 03.07.2009)**

What does one say about a dedicated academician, an astute clinician; a versatile professional well versed in Orthopedics, Physical Medicine and Rehabilitation and a great endearing colleague and friend!

Dr. Navnendra Mathur did his MS Ortho from SMS Medical College, Jaipur, in 1980. He then did his DNB in Physical Medicine and Rehabilitation in 1986. The following year he was selected for the prestigious IOA Johnson and Johnson Fellowship. After a stint as orthopedic specialist at King Fahd Hospital, Jeddah, Saudi Arabia he returned to join as Assistant Professor in Physical Medicine and Rehabilitation. He rose to the post of Professor and Head of Department, Physical Medicine; Director of the Rehabilitation Research Centre set up by his mentor Dr PK Sethi.

Dr. Navnendra was virtually obsessed with the study of spinal injuries and presented many outstanding papers on the subject at national and international conferences including the International Spine and Spinal injuries Conference at Indian Spinal Injuries Centre, New Delhi; International Society of Physical Medicine and Rehabilitation Medicine, Seoul, Asian Oceana Conference of Physical Medicine & Rehabilitation, Nanking, China and ASCON at Vietnam last year. He was also slated to attend the International Spine meet at Hong Kong this month. A life member of IOA, Dr Mathur has to his credit more than 25 publications in international and national Orthopedic and Physical medicine journals.

Born in November 1951, Dr. Navnendra had yet to complete 58 years. He passed away on July 3, 2009. He is survived by his wife Dr. Sushma, a CGHS GDMO, and two daughters.

